# Focusing a realist evaluation of peer support for paediatric mental health

**DOI:** 10.1007/s44192-023-00045-2

**Published:** 2023-10-05

**Authors:** Dean M. Thompson, Mark Bernard, Bob Maxfield, Tanya Halsall, Jonathan Mathers

**Affiliations:** 1 Institute of Applied Health Research, Murray Learning Centre, University of Birmingham, Room 239, Edgbaston, Birmingham, B15 2TT UK; 2 Forward Thinking Birmingham, Birmingham Women’s and Children’s NHS Foundation Trust, Birmingham, UK; 3 The Royal’s Institute of Mental Health Research, University of Ottawa, Ottawa, ON Canada

**Keywords:** Interviews, Paediatric mental health, Peer support, Realist evaluation

## Abstract

**Objective:**

Mental health problems are a leading and increasing cause of health-related burden in children across the world. Peer support interventions are increasingly used to meet this need using the lived experience of people with a history of mental health problems. However, much of the research underpinning this work has focused on adults, leaving a gap in knowledge about how these complex interventions may work for different children in different circumstances. Realist research may help us to understand how such complex interventions may trigger different mechanisms to produce different outcomes in children. This paper reports on an important first step in realist research, namely the construction of an embryonic initial programme theory to help ‘focus’ realist evaluation exploring how children’s peer support services work in different contexts to produce different outcomes in the West Midlands (UK).

**Methods:**

A survey and preliminary semi-structured realist interviews were conducted with 10 people involved in the delivery of peer support services. Realist analysis was carried out to produce context-mechanism-outcome configurations (CMOC).

**Results:**

Analysis produced an initial programme theory of peer support for children’s mental health. This included 12 CMOCs. Important outcomes identified by peer support staff included hope, service engagement, wellbeing, resilience, and confidence; each generated by different mechanisms including contextualisation of psychoeducation, navigating barriers to accessing services, validation, skill development, therapeutic relationship, empowerment, and reducing stigma.

**Conclusion:**

These data lay the groundwork for designing youth mental health realist research to evaluate with nuance the complexities of what components of peer support work for whom in varying circumstances.

**Supplementary Information:**

The online version contains supplementary material available at 10.1007/s44192-023-00045-2.

## Introduction

### Background

Mental health problems are a leading cause of health-related burden across the world, representing as much as a third of disability-adjusted life-years (DALYs) lost during childhood [1]. Between 2017 and 2021 in England, rates of probable mental health problems in children aged 6–16 increased from 11.6% to 17.4% [2]. Referrals have increased by nearly 60% over the same period. During 2019–20, 144,384 children were turned away—their referral was closed before treatment—and 64% of children referred to children’s services had not yet received treatment within the year despite the benefits of early intervention [3, 4]. The problem is magnified upon examination of regional variations. Thirty percent of people residing in the West Midlands (England) are in the most socioeconomically deprived quintile [5]. Twenty-six percent of children in the West Midlands live in relative low-income families [5]. Seventeen percent of people residing in the West Midlands, particularly in the most deprived areas, are from ethnic minorities [5]. Twenty percent of the West Midlands population lives in rural areas, and for those in industrial towns, there has been a decline in local jobs markets. The proportion of children aged 16–17 not in education, employment or training in the West Midlands is statistically higher than national average (5.5%) and as high as 10.3% in Shropshire and 8.5% in Birmingham [5]. The intersectionality of childhood inequalities in this milieu play out in health outcomes observed across the region. Through mechanisms like shame and withdrawal from social participation related to these socioeconomic circumstances, children in these communities may experience reduced access to services that improve health [6].

A report commissioned by all 11 Integrated Care Systems across the Midlands aimed to understand the reasons why the region had the lowest percentage of children accessing mental health services across England [7]. Key findings highlighted that, [1] as a region the West Midlands has complex processes for children accessing services, disadvantaging some children more than others; [2] that demand for mental health services outstrips supply, with demand expected to rise further due to the effects of the COVID-19 pandemic; and [3] there is a lack of prioritisation of continual service improvement. Recommendations from the report included the need to collate and share good examples of service user involvement in improving access to services; to work with service users to understand wider sociocultural needs to inform service provision; to develop a workforce strategy for eating disorder services; to evaluate different service models across the region; and explore the association between demographics and workforce on access and outcomes.

Peer support is widely regarded to improve hope for recovery, and access to mental health services [8]. Peer support involves people with lived experience of mental health problems supporting others in their recovery from mental health problems [9]. Peer support is part of the recovery movement that recognises the right and capability of people with mental health problems to participate fully in society, that they can recover *from* illness and lead productive fulfilling lives *in* recovery [10]. Here personal recovery is distinguished from clinical recovery in which people are encouraged to ‘return to normal’. Much of the history of peer support and peer support evaluation has traditionally focused on adults. However, recovery is policy in the UK for all [11], and efforts are increasingly oriented towards personal recovery through peer support in children [12].

Gopalan and colleagues [13] conducted a scoping review of youth peer support in the US [13]. They recommended that future research in youth peer support focus on specific mechanisms of influence, and in particular, whether matching between persons who use services and peer support workers’ characteristics influences treatment outcomes [13]. Realist evaluation is particularly suited to this line of enquiry, which aims to improve our understanding of how such complex interventions may trigger different mechanisms to produce different outcomes [14]. One existing realist evaluation aiming to understand the underlying mechanisms contributing to what works in peer support for children was undertaken in the Toronto-based Transitional Aged Youth program at LOFT Community Services [15, 16]. LOFT Community Services implemented peer support services alongside case management, mental health and housing support services for children and young people aged 14–26 years of age most of whom experience mental health and substance use problems. During the study, most procedures moved online due to COVID-19 pandemic public health restrictions, which incidentally may provide useful contextual information for how peer support works in a pandemic for older children and young adults. However, the study may lose some explanatory power as the impact of the pandemic begins to fade. In their realist evaluation, Halsall identified peer empathy, authenticity, openness, and similarity between peers as necessary components of peer support that contributes to service engagement [16].

Taking all of this in, peer support in *our* context in a post-lockdown West Midlands, when a sense of self-reported hopelessness in children has been on the rise during the pandemic [17], and where the supply ratio for children and young people’s mental health services has been constrained [7], presents a target for investigation to refine understanding of how peer support produces outcomes. Cumulation in realist research to improve services is typified by progressive problem shift, represented here by exploring the complexities of peer support in a socioeconomically deprived and ethnically diverse region amplified since the onset of the COVID-19 pandemic. An important first step in realist evaluation is to construct an initial programme theory to ‘focus’ the evaluation including determining the scope of data collection processes, and direction of analysis of context-mechanism-outcome configurations [18].

### Aim

The aim of this study was to explore professionals’ theories of how peer support services work in different children’s mental health service contexts to produce different outcomes in the West Midlands (UK) as a means of transparently creating preliminary data that may be used to inform further evaluation of peer support.

## Methods

A realist evaluation focusing study was conducted and is reported here with regard to RAMESES II Quality Standards for Realist Evaluation [18] (Additional file 1). A Patient and Public Involvement (PPI) Group was formed to contribute the conceptual development and research design of this project in accordance with UK Standards for Public Involvement [19]. The Group advised on the protocol, survey design, interview topic guide.

### Ethical approval

This study has been reviewed and approved by the Science, Technology, Engineering and Mathematics Ethical Review Committee at University of Birmingham (reference: ERN_21-1786). This study was performed in line with the principles of the Declaration of Helsinki.

### Setting

Children’s peer support settings included NHS mental health services, schools, charitable organisations, and private companies where a programme of intentional peer support is delivered in the West Midlands. The West Midlands region (Birmingham, Coventry, Dudley, Herefordshire, Sandwell, Shropshire, Solihull, Staffordshire, Stoke-on-Trent, Telford and Wrekin, Walsall, Warwickshire, Wolverhampton, & Worcestershire) has a population of approximately 6 million people and is home to one of the youngest cities in Europe, with children and young people (0–25) representing approximately 40% of the population. Each type of eligible study setting may cater for children and adults, for instance NHS children’s mental health services often support ages 0–25, though the focus of this study was children only (< 18 years) and participants were asked to reflect on this group of service users. Peers in services formally employing peers may refer to adult peers, though reciprocal peer support services refers to child peers. Though practice varies across services, peer support for children and young people typically involves another young person or slightly older adult with experience of mental health services (sometimes though not always the same service as the service user) delivering one-to-one or group-based mental health peer support. Peer support is delivered in isolation, alongside (or before or after) other more traditional forms of care typically depending on individual service user need. Further particulars of service settings are reported in Sect.  3.1 on survey responses.

### Programme theory

Initial programme theory in realist evaluation may ordinarily be derived from engagement with programme leaders (the purpose of this transparent preliminary data collection), or the extant literature, in this case borrowing from the adult peer support evidence base, which was used to inform discussion with participants on how peer support may work with professionals in the present realist-focusing exercise.

Gillard’s change model of adult peer support invoked Social Comparison Theory [20], Social Learning Theory [21], Bordin’s Trans-Theoretical Formulation [21], and Attachment Theory [23, 24] to articulate how peer support works by building a trusting relationship based on shared lived experience by triggering positive recovery role modelling, and community and service engagement [25]. In peer support between children, Social Comparison Theory would posit that peers role-model how children may think or behave [20]. Where the child attends to the peer’s words or actions that possess relevance and perceived value, the child may be motivated to internalise new more adaptive ways of thinking or behaving. Similarly, Social Learning Theory would posit that sharing lived experience normalises mental health problems, and when a comparison is made by the child whom perceives that their peer is in ‘a better place’, the child derives hope with the peer as their frame of reference [21]. Moreover, where children have trouble in forming trusting bonds, they may more readily relate to peers who demonstrate the requisite understanding to underpin a resilient therapeutic relationship [22, 23]. Indeed, these theories of how peer support works is common throughout the wider peer support literature [24].

### Sampling and recruitment

A preliminary, realist-focusing multi-site case study sample of professionals working alongside peers was recruited to capture a small albeit broad view of peer support across geographical locations and service type across the West Midlands, UK. As part of an asset mapping exercise to catalogue peer support assets in the West Midlands, each local NHS Trust and secondary school, and charities (identified via a Google search comprising term combinations of ‘peer support’ and regional localities) were approached to complete a survey about their peer support offer (see Sect. 2.5.1 on survey data collection). All respondents who provided their contact details and permission to be contacted for the purpose of recording an interview were invited for a follow-up interview.

### Data collection

#### Survey

A survey was administered to professionals working in services where peer support is offered to children to gather information about where peer support is offered in the West Midlands, to whom it is offered and how, including initial views on how they think peer support causes outcomes. The survey was developed in Jisc Online Surveys [27], and comprised categorical fixed response and open-ended free-text questions about [1] the respondent’s role and where they are based; [2] how peer support is delivered (content and modality), to whom, and what resources are in place to support this; [3] who peers are, how they are screened, trained and matched to other children, and [4] what outcomes are important and how these are generated by underlying mechanisms. The survey was piloted by two clinicians working in services where peer support is offered to children, who were asked to complete the survey and comment on usability and relevance of questions and response format.

#### Interviews

Qualitative semi-structured interviews were conducted with professionals working in services where peer support is offered to children to explore how peer support works, for whom and in what circumstances. Interviews were supported by a topic guide informed by the RAMESES II Project ‘starter set’ of questions [28]. The topic guide consisted of open-ended questions and prompts addressing variability in outcomes across children; how their local peer support offer may have caused those outcomes; how their peer support intervention may have changed their views on the objectives of peer support (to probe for participant reasoning about specific aspects of how peer support works); and how the participant would adapt their peer support offer to make it more effective to elicit experience of programme failure where mechanisms have not been triggered by the peer support intervention.

Interviews were undertaken by DT from 28th April to 12th July 2022. DT is a chartered psychologist with experience of conducting qualitative research, consulting on research design including realist research studies, and delivering psychological therapies independently of the sampled services. Interviews were audio-recorded and transcribed clean verbatim and anonymised by a professional transcriber for data analysis.

### Data analysis

Categorical survey data were tabulated, and free-text survey item responses underwent simple content analysis by DT [13], except for the survey item designed to elicit CMOCs, which fed into the realist interview analysis. The realist epistemological position holds a theory of causal explanation based on generative principles. Ontologically speaking, realism assumes that regularities in patterns of social activity or interventions are generated by underlying mechanisms that may be triggered in particular contexts [14]. Methodologically, this analysis seeks to explain the configurations between context, mechanism, and outcome (CMOC) to explain how contextual components of peer support (C) work to produce an outcome (O), because of an underlying mechanism (M).

Mechanisms in realist research are often challenging to parse even for the experienced realist evaluator. At their core, mechanisms may refer to the choices or capacities resulting in regular patterns of social behaviour. So, we may hypothesise based on existing non-realist theory that a social peer support intervention may be introduced to counter problematic social causal mechanisms like stigma and barriers to inclusion and diversity in services. At the core of any realist analysis is the imperative to uncover whether a programme (of peer support) has nullified the problematic mechanisms responsible for the original problem. In this case, that problem would appear to be focused on a lack of local service engagement contrasted against the potential for a more diverse workforce acting out alternative causal mechanisms to mitigate this [7]. During analysis, we adopted the realist principle of embeddedness to guide analysis of programme systems. Analysis rejected the notion that programmes in general (including peer support) are solely targeted at service users and that an effective intervention only changes individual service user behaviours or thoughts. The realist principle of embeddedness fits well with peer support, which may target *and benefit* the service user, peer, mental health service and their staff, and the wider community. Peer support programmes may be designed to be uni-directional (the peer supports a service user), or mutual (support between two peers/service users) [29]. This analysis considers outcomes for both child and peer across social interactions that may be one-directional or mutual, and considers propositions accounting for micro and macro social processes.

Realist interview data were imported into QSR International’s NVivo version 12 software [30], and organised using Gilmore’s transparent approach to realist analysis [31]. DT read each transcript line-by-line. Coding into nodes occurred where a CMOC was observed (or part of a possible CMOC), and child nodes created where theory was revised. Nodes were also created where participants referred to context or outcomes where potentially relevant to CMOCs noted elsewhere in their interview transcript. A memo was linked to each node articulating decision-making underpinning any theory refinement. During the coding process for each new interview transcript, memos were reviewed, and CMOCs were combined or divided where resemblances or differences between them were observed. The resulting CMOCs and associated illustrative quotes from the interview transcripts were reviewed by all other members of the study team (MB, BM, TH & JM) including one member with experience of realist evaluation (TH) for agreement.

## Results

### Survey responses

Fourteen participants included a CEO (n = 1), commissioner of services (n = 1), service manager (n = 2), recovery/project leads (n = 5), clinical psychologists (n = 3), teacher (n = 1), and community moderator (n = 1). Service setting and programme details are presented in Table 1.

**Table 1:** Service settings and programmes

#	Service type	Location	Modality*					Programme
			F2F	Online	1:1	Group	Mutual pairs	
01	Charitable organisation partnering with NHS	Currently North Birmingham—plans to expand to cover WMCA	X			X		Four-week co-designed interactive peer support. Sessions focus on themes (self-esteem, resilience, confidence, identity, stress, peer pressure) with journal entry and 12-month follow-up. Informal peer support between children encouraged between appointments and after completion of programme
02	Local authority (public health)	Walsall	X	X		X		Not reported
03	NHS CAMHS (Child and adolescent mental health services)	North staffs and Stoke	X	X	X		X	Peer-delivered talking therapies
04	NHS, Early Intervention in Psychosis Service	Birmingham	X		X	X		Sharing of lived experience, including treatment experience to provide hope. Organisational involvement through providing patient voice in service team meetings, recruitment interviews and liaising with other organisations
05	NHS mental health service	Solihull	X	X		X		Patients are involved in co-facilitating some groups (psychoeducation, carers & relaxation/self-care/mindfulness groups), offering input in quality improvement initiatives and recruitment interviews
06	NHS Early Intervention in Psychosis / Community Recovery teams	Coventry and Warwickshire	X	X	X			Peers use their lived experience and recovery to build a relationship with the person they are working with to build hope and a sense of connection. They develop recovery goals, and use wellness and recovery action plans and problem-solving
07	NHS Forward Thinking Birmingham	Birmingham	X	X	X	X		Peer support focusing on physical health and wellbeing; recovery groups; and support into social inclusion activities, education, training and employment
08	Charitable organisation partnering with schools	Walsall/Wolverhampton/Dudley and Sandwell	X		X	X	X	Training (including certificates) and ongoing monthly support
09	Peer Mediation		X			X		Peer support guided by mediation resource packs
10	Charitable organisation	Coventry and Warwickshire	X		X	X		Peers use their lived experience as the basis of support, complemented using 'walk and talk', focus on the interests and needs of the service user (i.e., cooking, safety planning, setting goals & helping to empower & build confidence)
11	Secondary school	Handsworth, Birmingham	X		X			Peers who listen, advise, and signpost
12	NHS Eating Disorder Service	Birmingham	X	X	X	X		Peer support focused on recovery and relapse prevention, including co-facilitation of clinical group work
13	Private company	West Midlands	X	X		X		Deliver training around how to get children and young people involved at all levels in the running of service; and training around the specific mental health needs of disempowered and diverse groups (LGBTQ + , neurodiverse & people of colour) as well as diagnostic specific training (Eating Disorders & Personality Disorder)
14	Private company	Online (West Midlands)		X		X		Anonymous and moderated online peer support community, including articles written by peers and professionals to help inform and educate on a range of mental health and wellbeing topics. Moderators signpost to in-house Emotional Wellbeing Practitioners and counsellors for 1:1 support

^*^ F2F (Face to face); 1:1 (One to one)

### Initial programme theory

**Fig. 1:**
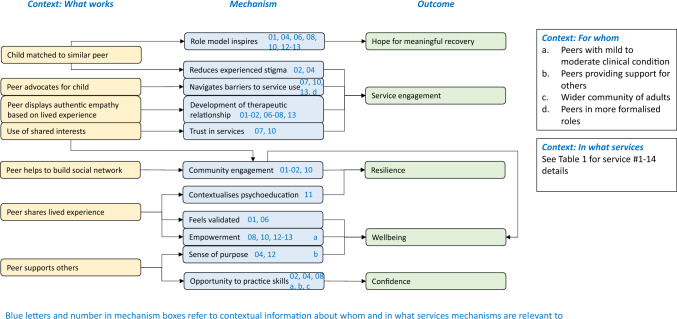
Youth peer support initial programme theory

Analysis of 10 preliminary interviews with peer support service staff produced an initial programme theory of peer support for children’s mental health (Fig. 1). This web of CMOCs takes on a complex presentation in keeping with its embryonic stage of development and limited preliminary data from staff only across a range of sites with subtly (or more obviously) different contextual implementation like peer support delivered as delivered in health services contrasted against delivery in schools. This includes 12 context-mechanism-outcome configurations (CMOCs). ‘Context’ of what works is represented by individual components of the peer support intervention that trigger a mechanism to elicit an outcome. In Fig. 1, each mechanism contains blue numbers referring to contextual information about what type of services are relevant to the mechanism. These should be cross-referenced with Table 1. Not all mechanisms contain blue letters to refer to contextual information about who mechanisms are relevant to (like demographic subgroups) as participants were not always able to, or actively chose to refute and distinctions within their service.

### Individual context-mechanism-outcome configurations (CMOCs)

In this section, explanatory summaries underpinned by illustrative quotes are provided for each individual CMOC, organised by Outcome.

#### Hope

##### Presence of matched peer, role-model of recovery

**Fig. 2:**
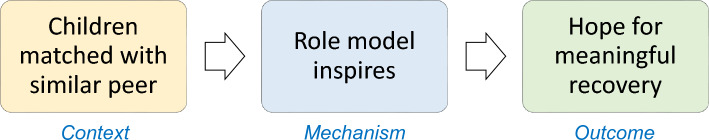
CMOC 1

When a child is matched with a similar peer (C), they develop hope for personal recovery (O) because they have been inspired by a similar peer modelling recovery (M) (Fig. 2).

Hope for recovery predominated participant accounts of the value of peer support. They considered this to be an outcome in the context of peer support, with more distal clinical outcomes like anxiety and depression) compartmentalised as a consequent outcome that some though not necessarily all children may derive from more traditional clinical service provision alongside peer support. Staff consistently and deliberately avoided being drawn on what distal outcomes may be generated by hope (acting as context) because ‘personal recovery’ was deemed to be a uniquely independent experience and not necessarily intended as a necessary outcome of peer support as part of a wider treatment package. Peer support worked well when children were matched to similar peers who would embody the possibility that recovery is possible:

*“People perhaps don’t always understand the role of a peer and expect them to go about sharing all the difficulties in their life, but they are not there to share all the difficulties, they are there to model hope.”* [#13]

*“I think it’s always helpful to have someone who you know has been there and has experienced this and has found this is harder than perhaps you’re finding it, who almost is your model. So, it’s easier to do something when someone is modelling it for you, and they’re wearing their experience on their sleeve of right I have been here, ‘I have done this, I know how it feels to do this, but it is possible’. So again, instilling that hope that this feels really hard, and this is overwhelming right now, but it is possible.”* [#12]

Here, the nature of the similarity is left unspecified by the professional, other than to allude to a past albeit similar overwhelming hardship, that may be focused on mental health symptoms, sociodemographic intersectionality, or challenges when attempting to engage with services (more on this to follow). Of note in the above illustrative quote, is the implied use of downward comparison ‘[the peer] found this is harder than perhaps you’re finding it’ which was not generally elicited in other interviews with professionals.

#### Service engagement

##### Peer advocates for child, navigating barriers to service

**Fig. 3:**
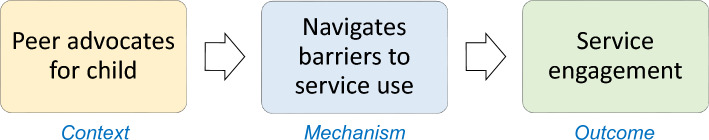
CMOC 2

When a peer advocates for a child (C), service engagement increases (O), by navigating barriers to accessing or continuing to engage with services access (M) (Fig. 3).

One way in which peer support could lead to greater service engagement and access to care was through peers advocating for children to help them to access adult services when transitioning from childhood:

*“When they are transitioning from children’s mental health services to adult mental health services. So again, that’s where that waiting list remit might come into it or those other areas where they are transitioning, we can go in and act as advocates and support them.”* [#10]

Children’s perception of services as ‘oppressive’ was described as a problematic mechanism preventing service engagement. Participants indicated this could be interrupted by the introduction of peers to support children in this context to change children’s reasoning in favour of service attendance where a similar peer may be able to mitigate undesirable components of traditional care. Peers may help children to overcome perceived institutionalisation and oppression experienced in clinical settings, with peers in a position to help children in these services, in part by bypassing other clinical care within these services entirely:

*“I think there’s possibly an issue there where there’s the same groups of people who might not want to access services because they are traditionally seen as oppressing them in some way, and then peer support could be a really viable alternative.”* [#13]

However, this mechanism may not be triggered in service contexts in which peers are subsumed by clinical systems and teams and may have limited capacity to advocate for the children they work with:

*“We definitely see it with statutory services, there’s this idea of institutionalisation where a peer might start to align themselves more with the team… but due to the way that it’s perhaps set out or functions within the system it doesn’t always keep that same transformative element that it could hold to it.”* [#13]

##### Child matched to similar peer, mitigates experienced stigma

**Fig. 4:**
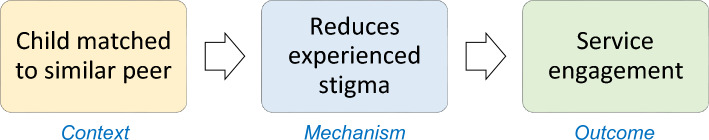
CMOC 3

When a child is matched with a similar peer (C), service engagement increases (O) by reducing experienced (instigated by others) stigma (M) (Fig. 4).

Staff recognised the stigma experienced by children and how it may be realised in their interactions with others in a way that could inhibit children accessing care. Furthermore, peer support was described as a way to match children to similar peers (by age, religion, gender, lived experience of mental health problems) so that mental health interactions could be normalised or effectively disguised as an ordinary social interaction:

*“A young Muslim man wouldn’t really want to come for a walk with me if I was his care co-ordinator, if I was his nurse, because his friends would see him and say, “What are you doing with that middle aged white woman?” So, there’s something around the stigma, so if we can try and match them to a younger person that they might be more likely in real life to have a relationship within their social network that’s helpful too.”* [#04]

##### Authentic empathy from lived experience, therapeutic relationship

**Fig. 5:**
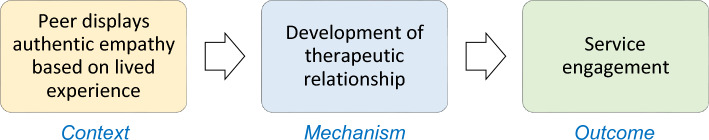
CMOC 4

When peers display empathy (C), service engagement increases (O), reinforced by a developing power-balanced therapeutic relationship between the child and their peer (M) (Fig. 5).

Mechanistically, the potential for a traditional therapeutic relationship between service user and clinician is not refuted, though is replaced by a particular therapeutic relationship that is characterised by a balanced power dynamic. In our interviews, staff suggested peers possess a more authentic empathy through their first-hand lived experience, with a willingness to suffer with the child they are supporting to maintain engagement with the service in which they are situated:

*“They are more there because they have got this authentic empathy of being able to say I have been in that place where it’s like I understand how it can feel for people to look at you in this way, or to not believe in you, or for you yourself to feel that there’s not a road forward, but I am here to explore with you the ways in which we can construct this way forward and I will walk with you alongside it together.”* [#13]

The idea of ‘walking alongside’ here was invoked not to suggest literally walking with the child in the community (although this was referred to previously in this analysis). Rather, it is used to denote compassion as in the Latin root compati, ‘to suffer with’.

Openness of peers lends itself to perceptions of authenticity, which balances power between child and peer, enabling the child to let their guard down so that they are better able to engage with their service beginning with their peer. However, one caveat was noted—remunerated peers may lessen the appearance of authenticity:

*“This idea of being able to have a mutual and reciprocal relationship in that sense, being able to sit with someone and the power balance being as close to equal as possible. Obviously with intentional peer support someone is paid, so that has to be addressed, but I think part of the authenticity is being open and honest and not holding bits of information back because they see the young person as not being able to handle it, and with these conversations I think there’s maybe a feeling of having to be a rescuer or the protector of the young person, and I guess be able to be authentic and be ourselves, allow the young person to be themselves, maybe let them bring their guard down”* [#13]

##### Use of shared interest between child and peer, Trust in services

**Fig. 6:**
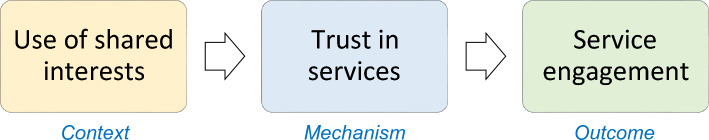
CMOC 5

When peers use shared interest to engage with children (C), service engagement increases (O) because children feel that they can trust services (M) (Fig. 6).

Staff noted that for the use of shared interests to improve service engagement, it was important that children were motivated and that any opportunity to engage in shared interests together is funded. Trust in this case may be generated by a perception that recovery is not purely medicalised, the use of medication highlighted by this member of staff:

*“When we break it down and look at some of the groups that aren’t engaging well with that we tend to find a lot of young males are not interested in it, we find that don’t want to engage in anything physical health and wellbeing. The feedback can be either I don’t need it, not interested in it. There are some things where they say I don’t have the opportunity, there’s nothing in my area, I can’t afford it, I can’t do that, and I think so there are those groups… we have got a couple of young black peer workers that are in the team who are doing some activities around music… What that’s leading into is a bit of more of a trust in the service and a bit more of a trust in actually you’re not just there to pump me full of meds, you’re not there just to do this to me, that to me, there’s an opportunity there… that has helped with a wider engagement, it’s something they’re interested in, the peer worker is interested in themselves.”* [#07]

#### Confidence

##### Support others, develops skills

**Fig. 7:**
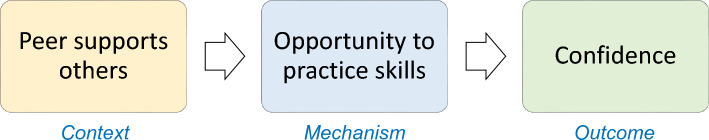
CMOC 6

When a peer supports others (C), their confidence improves (O), because they have had the opportunity to practice and develop skills (M) (Fig. 7).

The potential for peers to develop confidence via skill development was situated within a social context that values career (as a more distal outcome), although the social interaction of children and peers could trigger social skill development in peers to generate confidence as an end in and of itself:

*“I think there’s also something about the peer workers themselves gaining skills and confidence, and to be able to get a foothold on a career ladder, see themselves differently, so the peer worker and the person they’re working with gets a benefit.”* [#02]

*“Identify some young people for whom peer mentoring might be a really positive social learning tool and a positive thing for them to do to give them confidence.”* [#08]

#### Resilience

##### Build social network, engage with community

**Fig. 8:**
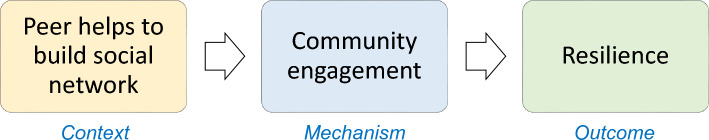
CMOC 7

When a peer helps to build a child’s social network (C), the child develops resilience to recover from setbacks (O) because they can engage with individuals or assets within their community (M) (Fig. 8).

Staff acknowledged the isolation associated with mental health problems, compounded by the potential for disrupted community engagement due to the COVID-19 pandemic. In peer support, peers can actively help children to (re)build their social network. In contrast to the value of peer support as an intervention to help children to engage with clinical services, staff also described how peers may help to build awareness of assets within their community that they would feel able to engage with going forward so that they felt that they no longer required clinical services:

*“To continue that support without us, they access like I was saying earlier about social isolation, they have built those networks, they have brought those links within the community or the professionals or identify links within their own home and family and friends. So, they built that resilience to say ‘actually I don’t need you anymore’.”* [#10]

##### Peer shares lived experience, contextualise psychoeducation

**Fig. 9:**
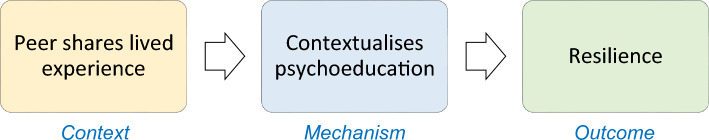
CMOC 8

When the peer shares lived experience in a context where psychoeducation is delivered by the peer or clinicians (C), the child’s resilience may be improved (O) by understanding how to apply the psychoeducation in practice (M) (Fig. 9).

Professionals suggested that peers could provide an example of how children can help relate learned models of resilience to their day-to-day lives:

*“Resilience is something that might be talked through sessions on transactional analysis, of part of a PHSE [Personal, Social, Health and Economic education] programme, but actually there might be some pupils that they just need a bit more of a reminder of what that looks like. The scenarios that we have looked through in class they aren’t enough, they need to actually relate it to their here and now, their personal experience, and that’s where this [peer support] comes in, it complements it. It’s all part of that bigger picture.”* [#11]

#### Wellbeing

##### Peer shared lived experience, sense of validation

**Fig. 10:**
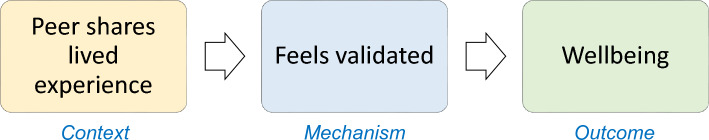
CMOC 9

The act of supporting others (C) improves peer wellbeing (O), by helping peers to feel validated (M) (Fig. 10).

One service staff member describes sharing lived experience as cathartic when enacted between similar peers due to the depth of their shared understanding. Here, peer support is contrasted against clinician-delivered care.

*“I think it’s [sharing lived experience] a release mechanism. I think it’s a way that firstly it’s acknowledgement but on a deeper level. It’s rather than somebody just sitting there and going oh that’s interesting or oh I’ve not had that before, or oh wow, it’s yeah, I get you, I understand. It’s that depth of understanding I think that comes from it. It’s acknowledgement, it’s being seen and being heard.”* [#01]

This sense of validation was also triggered by the work context and the service clinicians within it—the act of peers sharing their lived experience as part of, or alongside of a clinical team (that may in some circumstances have previously supported the peer) providing a sense of community belonging beyond any prior illness identity:

*“There’s also an aspect of the impact of this on peer workers themselves, so for example when people talked about the interview process afterwards what people were saying was that to be able to have their experience of psychosis and their recovery valued, to have it as something that was there to be talked about in a really positive way was such a different experience to how they have approached working interviews before where they have had to hide that, and there’s been a degree of shame about that as well. So to be in an interview when people are positively looking for that, and want to talk about that, again for them it’s something really positive.”* [#06]

##### Peer shares lived experience, feels empowered

**Fig. 11:**
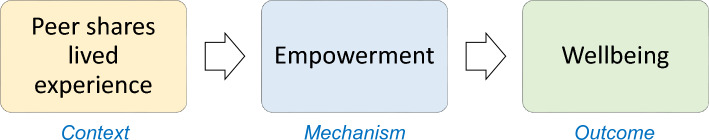
CMOC 10

When a peer shares information about their experience of a problem common to the child and peer (C), their wellbeing improves (O) because they have been empowered to control their own recovery journey (M) (Fig. 11).

Participants described empowerment as a programme mechanism that can be contrasted against disempowering ‘traditional’ clinical care that may have limited children’s control over their recovery. When peers are employed in a role that enables them to share their experience of a mental health problem, the role provides a sense of recognition or affirmation that the peer’s experiences and contributions are valid and worthwhile. This capacity effectively authorises (empowers) them to identify their own definitions of personal recovery that is meaningful to them to provide control over their own recovery:

*“It is such a contrasting role compared to I guess a lot of traditional in inverted commas mental health roles in it’s empowering, drawing upon that lived experience to validate… and looking at allowing that individual to define what recovery is for themselves.”* [#13]

However, in the context of eating disorder services, staff located empowerment as displaced from the child to their parent:

*“We do need to empower them, and we do need to disempower the eating disorder when actually it’s making decisions for the child. When the child is out from the grips of the eating disorder then we can empower them to make decisions. But when they are that unwell, they just make decisions to not eat, and that’s when we have to empower parents to do that for them.”* [#12]

##### Peer supports others, sense of purpose

**Fig. 12:**
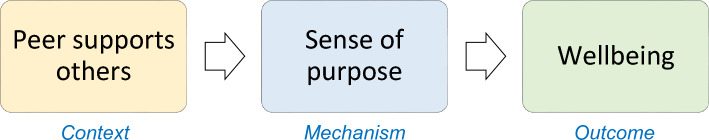
CMOC 11

When a peer support worker helps other children (C), their wellbeing improves (O) because they develop a positive sense of purpose or meaning to their own experiences (Fig. 12).

Staff relayed common narratives in their peers’ attempts to generate a positive understanding of the mental health challenges they had experienced, including for example, reducing feelings of guilt by supporting others:

*“I would hope going back to the CHIME [Connectedness, Hope, Identity, Meaning and Empowerment] model *[32]* they get some sense of meaning. Many of my patients have said to me that… “I have been here for so long I have just lost so much time and it just feels like what was the point?” And all of this, and I wonder if this helps them feel like actually there was some meaning to this, at least there was something that I could say that it was there was a point to it, so I was unwell, my eating disorder hijacked my life, and I lost this massive chunk of my life, but I am able to create some meaning from it by using it to support somebody else to make their recovery journey, and that makes it worthwhile. I don’t have to feel guilty about the fact that I left that bit behind.”* [#12]

However, it is acknowledged that working in the peer role poses risk to peer wellbeing where the work triggers latent mental health difficulties:

*“But then on the other end we have got one peer support worker who he is one of the older ones, he has had to take time off because he’s really struggling doing engagement with people because it’s triggered his mental health condition and he’s not very well at the moment. So, it’s a real challenge, it is for anybody, but I think particularly this group because it’s ongoing recovery and it is ups and downs.”* [#04]

##### Use of shared interest between child and peer, community engagement

**Fig. 13:**
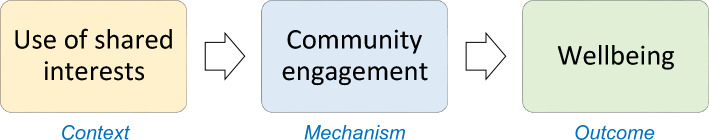
COMC 12

When peers use shared interest to engage with children (C), children’s wellbeing improves (O), because they have developed a social network within their community (M) (Fig. 13).

Staff recognised that peers may tap into their shared interests to help children to engage with community assets in a way that benefits their wellbeing by engaging with community assets that are associated with social determinants of good health and wellbeing:

*“We have some flexibility to do other drop-in one to one kind of support, and they will then network with volunteers, some of whom will be in the age range, some of whom will have specialist skills in things like football or fishing or whatever… because then we can instil a public health social determinants kind of way, not just in a you’re going to have counselling because you’re a bit depressed.”* [#02]

*“It will also be the connectivity, so it’s a community asset-based approach… because [the area] is very focused on community, and there are very definite neighbourhoods to leverage some of that community support and to connect people… So, if we’re talking about more public health wellbeing approach to connect people through peer working into the communities and how to access local support and friendship groups and those kind of things are going to keep people well, not just fix what ails them now.”* [#02]

Peers were described as a relevant culturally competent point of contact (contrasted against other staff who may only signpost children to community assets), uniquely placed to support children in their shared interests:

*“I think the fundamentals remain the same wherever you are, whoever you’re with, whatever you’re doing actually. There are some things that you need to be mindful of and you need to adapt to. So, for example quite often I think it’s about placing the right people within the right areas to do that specific thing. So, we run a creative arts programme, we have also done the community, we have supported the community dance group with the first pilot of the young people’s programme. I have not done either of those because I haven’t got the right skills to be working with young people, but we have got two people on our team who are trained to work with young people, that’s what their background is, that’s what their experience is, and they also have got lived experience of being a young person and having a mental illness, which is really important. It’s about knowing your people and putting the right people in the right place as much as anything, I think. I don’t run the creative arts programme because I wouldn’t have a clue, I couldn’t. So, we have got somebody who specialises and who is a fine artist and has done artwork within her local community and all her training is in art and set design, and she delivers that with her lived experience of mental illness as well. I think I would say that for us that’s the only difference, right people right place, but everything else remains.”* [#01]

## Discussion

The preliminary data reported in this paper provides transparency in the decision-making process of developing an initial programme theory to contribute to the development of cumulative realist evaluation of peer support for children by focusing the scope of context-mechanism-outcome configurations [18]. This study proposes an embryonic initial programme theory based on a preliminary survey and interviews with professionals across healthcare and education settings. The professionals interviewed in this study identified five peer support outcomes, presented here in the initial programme theory (Fig. 1). The outcomes foregrounded by participants were hope for recovery and service engagement, which tallies with existing research with adults and children in other peer support settings [25]. There was also evidence of improved resilience, confidence, and wellbeing. These outcomes were consistently targeted across the variety of peer support models and populations they support, though professionals reiterated throughout that personal recovery is very much about what is important to individual service users, and that what these outcomes look like to different children will vary significantly from person to person. This is in keeping with Halsall’s realist evaluation of peer support at the Toronto-based LOFT Community Services for children and young people aged 14–26 years [16], which posited an overarching ‘recovery’ outcome generated by mechanisms familiar to the present evaluation (including participation in social activity, which is roughly synonymous with community engagement). Divergences between the two realist evaluations will have been caused by the different intervention context inherent in each study, resulting in subtle differences in outcome focus (participants in the present paper highlighting experienced stigma rather than self-stigma as in Halsall’s evaluation, while both are acknowledged in Gillard’s work with peer support between adults) [25]. The underlying reason for the differences here may be due to design (the preliminary ‘focusing’ purpose of the present paper with staff only less able to reflect on individual stories of self-stigma) rather than contextual programme variation.

Realist methodology represents a fitting approach for exploring what intervention contexts produce these outcomes and for whom. Professionals perceived that service engagement increases in response to careful matching of peers to children by reducing experienced stigma from others, advocacy by navigating barriers to services, use of shared interests between the peer and child to improve trust, and authentic empathy by developing a therapeutic relationship between child and peer. In contrast to these myriad routes to service engagement as an outcome of peer support, professionals articulated that hope was singularly generated by peers sharing their lived experience which may inspire other children. The mechanisms and outcomes reported in this paper resemble Gillard’s change model of adult peer support interventions [25, 26], albeit reformulated from a realist perspective [14]. In contrast to the adult peer support literature in which advocacy may be seen as counterweight to empowerment which peer support should aim to engender, advocacy may have been seen a more positive light by participants working with children. Whether this is due to differences in children and young people’s nascent development and needs may be one are for further study.

Indeed, some caveats to how peer support interventions generate outcomes in different contexts were revealed by professionals, indicating when mechanisms may not be triggered when working with peers in particular service settings. In eating disorder services and when children are experiencing acute mental health problems, the problem of power imbalance between service user and clinical professionals (M) is not countered by triggering empowerment (M) due to staffs perceived risk of harm (disordered eating). However, the capacity for empowering children was nonetheless triggered vicariously by displacing power from clinical professionals to parents to manage wellbeing (O).

This study was concerned also with the peer supporter and the potential benefit derived from inhabiting and working within the role, for instance, peer support worker skill development benefitting their confidence with the potential for more distal outcomes for further education and employment. However, working with people experiencing distressing mental health problems can itself be challenging for professionals [33], and on this evidence, peers alike. The risk of peer worker relapse was acknowledged in our interviews with staff. However, the use of screening of prospective peers was limited in the study services. One potential ‘abuse’ of recovery-oriented practices described by Slade concerns allowing contribution to society only after a person is recovered, which has been described as a ‘major injustice’ [34]. Moreover, provided that the peer can fulfil the peer role and not functionally impaired by severe distress, the use of indicators of mental health stability may fall foul of disability and employment legal protections. Thus, the lack of screening reported by staff in this study fits within the legal and recovery-oriented framework prevailing in the UK, notwithstanding the likelihood that the stress of working may be preferable to the challenges of prolonged unemployment [9].

The effect of peer support on improved service engagement was welcomed across the professionals interviewed in this study. This is consistent with existing evidence [16]. However, the local challenge of demand outstripping capacity in children’s mental health service provision in the West Midlands [7] risks programme failure due to ‘inappropriate contextualisation’ (where interventions do not work due to inhospitable contexts perpetuating problematic mechanisms). Namely, the use of peer support to increase service engagement may risk building up further unmet demand for support which could risk burnout or moral injury of peers employed within the healthcare system [35, 36]. A recovery orientation underpinned by peer support is not justification for cutting service provision [34]. Professionals interviewed in the study also refuted programme mechanisms leading to increased service engagement where children have the resilience to maintain their mental health recovery in their community using the community assets available to them while reducing service engagement.

The perennial debate on the relative merits of casual vs more formal professionalised peer support worker roles is broached again in this study and was similarly polarising [37]. More specifically here, children’s capacity to navigate barriers to services may not be triggered by peer support where peers may be perceived by children as being aligned with the clinical team. This notion was also refuted by one interviewee who argued that children are likely to pick up on peers’ affiliation with clinical services regardless. However, it would be challenging to assess this even with further research with children and peers for whom service engagement is wholly deterred at the point of entry (rather than maintaining engagement).

The theory explored in this paper fits with processes set out in Leamy and Bird’s conceptual framework of personal recovery for adults which was raised in conversation with participants in this study [32]. For example, when discussing children from UK ethnic minorities, professionals placed emphasis on matching peers to children (based on religion, ethnicity, gender, and age) to improve engagement by reducing experienced stigma associated with the interaction between peer support provision situated in the community. Similarly, Leamy and Bird highlight the value of belonging to a particular cultural group or community which is salient here particularly in relation to the age gap between child and peer (although the exact nature of the effect of any age gap by year is not explored in this paper) [32]. Where the present paper departs—in placing peer support at the centre of enquiry—is a multifaceted appreciation of peer support that is explicitly interconnected with the other processes of personal recovery, namely inspirational (peer) relationships leading to hope for the future; overcoming stigma by matching children with similar children (peers); and inhabiting the (peer) supporter role to empower young people by validating their experiences and control over their care. Crucially, the realist approach beginning here attempts to do away with Leamy and Bird’s finding that the *recovery journey* can be a process of *trial and error*, throwing intervention component context into the wind and hoping it sticks. Such is life we might say. However, our responsibility as healthcare professionals, teachers, researchers, anyone working to improve children’s mental health is one of continual service improvement. Here we call for more research designed to uncover what works for whom and in different circumstances, and a little less trial, and a little less error.

### Limitations

Though professionals in this study did raise wellbeing, hope and resilience as important outcomes generated by peer support, interviews did not draw out these outcomes as potential distal outcomes generated by service engagement. Given all that we know about traditional clinical care in mental health services, there is extensive evidence of some effect of peer support on these outcomes as well as other more traditional clinical outcomes. This omission was in part due to intervention context moving from peer support to the more traditional clinical intervention, which was not the focus of this study. However, this may not exclusively be the case. For instance, inclusive peer support culture and practices in organisations may further influence outcomes, including the potential impact for the presence of peer support workers to have an impact on clinician’s perspectives on the possibility of recovery for some service users.

Clinical psychologists interviewed in this study, will typically have been trained in and routinely work from a cognitive behavioural stance. Thus, much of what was discussed concerned cognitive and behavioural theory potentially to the exclusion of other relevant theory. There are two counterarguments to this as a potential limitation from a realist perspective. First, realist evaluation seeks not to generalise to every context where it may be inappropriate to do so. Rather, a realist approach is concerned with evaluation towards service improvement in a particular context. Therefore, if clinical psychologists in the UK tend to favour a cognitive behavioural approach and way of formulating service user experience, then there may be reasons for this position, and the realist evaluation therefore explores what is relevant to this context. Second, this preliminary realist data collection with professionals was developed to explore the views of a range of professionals, including professionals who are not psychologists who will not necessarily have been trained in the cognitive behavioural tradition.

Exclusively interviewing staff may not represent an appropriate sample for determining where peer support does not quite work (to better understand how peer support could work or how to better target implementation) due to organisational affiliation and a preference to avoid criticising peers or their service. Furthermore, ineffective interventions may result in children disengaging with services to the extent that staff do not always have the opportunity to learn why things are not working for some people in some circumstances. However, this study was not intended as a complete realist evaluation, rather a transparent approach to publishing details of initial programme theory development. Moreover, it is common practice in realist research to initially work with the professionals who organise programmes, because they are knowledgeable about the intentions behind their programme [14]. Nonetheless, further research ought to engage with service users directly in a thorough realist evaluation to understand further what may *and may not* be working and why [13, 16].

## Conclusions

The data outlined in this report lays the groundwork for designing realist research to evaluate with nuance the complexities of what components of peer support work for whom in varying circumstances. Peer support may represent a promising investment where workforce development in more inclusive services is warranted as in diverse communities in the West Midlands of the UK [7]. Moreover, cumulative realist evaluation of such interventions in unique circumstances where implementation issues arise may help evaluators to explore different models of peer support delivery, and the association between demographics and workforce on service access and outcomes.

### Supplementary Information

Below is the link to the electronic supplementary material.

Supplementary file 1: RAMESES II reporting items

## Data Availability

The datasets used and analysed during the current study are available from the corresponding author on reasonable request.

## Abbreviations

CHIME: Connectedness, hope, identity, meaning and empowerment
CMOC: Context-mechanism-outcome configurations
DALYs: Disability-adjusted life-years
