# Supplementary material for: Focusing a realist evaluation of peer support for paediatric mental health
Source: Discov Ment Health. 2023 Oct 5;3(1):18. doi: 10.1007/s44192-023-00045-2 (PMC10555983; doi:10.1007/s44192-023-00045-2)
Supplement: Supplementary file 1 — RAMESES II reporting items [file 44192_2023_45_MOESM1_ESM.docx]

Additional file 1. RAMESES II reporting items

|  |  | Section(s) in document |
| --- | --- | --- |
| TITLE | |  |
| 1 |  | Title |
| ABSTRACT | |  |
| 2 |  | Abstract |
| INTRODUCTION | |  |
| 3 | Rationale for evaluation | 1.1. Background |
| 4 | Programme theory |  |
| 5 | Evaluation questions, objectives and focus | 1.2. Aim |
| 6 | Ethical approval | 2.1. Ethical approval |
| METHODS | |  |
| 7 | Rationale for using realist evaluation | 1.1. Background |
| 8 | Environment surrounding the evaluation | 2.2. Setting, 3.1 Survey responses |
| 9 | Describe the programme policy, initiative or product evaluated | 2.3. Programme |
| 10 | Describe and justify the evaluation design | - 1. Background |
| 11 | Data collection methods | 2.5. Data collection |
| 12 | Recruitment process and sampling strategy | 2.4. Sampling and recruitment |
| 13 | Data analysis | 2.6. Data analysis |
| RESULTS | |  |
| 14 | Details of participants | 3.1. Survey responses |
| 15 | Main findings | 3.2. – 3.3. IPT, CMOCs |
| DISCUSSION | |  |
| 16 | Summary of findings | 4. Discussion |
| 17 | Strengths, limitations and future directions | 4. – 4.1. Discussion, Limitations |
| 18 | Comparison with existing literature | 4. Discussion |
| 19 | Conclusion and recommendations | 5. Conclusion |
| 20 | Funding and conflict of interest | Footnotes |
